# The Posterior Cricoarytenoid Muscle Is Spared from MuRF1-Mediated Muscle Atrophy in Mice with Acute Lung Injury

**DOI:** 10.1371/journal.pone.0087587

**Published:** 2014-01-31

**Authors:** D. Clark Files, Kunhong Xiao, Tan Zhang, Chun Liu, Jiang Qian, Weiling Zhao, Peter E. Morris, Osvaldo Delbono, Xin Feng

**Affiliations:** 1 Internal Medicine-Pulmonary, Critical Care, Allergy and Immunology, Wake Forest School of Medicine, Winston-Salem, North Carolina, United States of America; 2 Wake Forest Critical Care Translational Research Center, Wake Forest School of Medicine, Winston-Salem, North Carolina, United States of America; 3 Department of Medicine, Duke University, Durham, North Carolina, United States of America; 4 Internal Medicine-Gerontology, Wake Forest School of Medicine, Winston-Salem, North Carolina, United States of America; 5 Department of Radiation Oncology and Brain Tumor Center of Excellence, Wake Forest School of Medicine, Winston-Salem, North Carolina, United States of America; 6 Department of Otolaryngology, Wake Forest School of Medicine, Winston-Salem, North Carolina, United States of America; Institut de Myologie, France

## Abstract

**Background:**

Skeletal muscle wasting in acute lung injury (ALI) patients increases the morbidity and mortality associated with this critical illness. The contribution of laryngeal muscle wasting to these outcomes is unknown, though voice impairments and aspiration are common in intensive care unit (ICU) survivors. We evaluated the intrinsic laryngeal abductor (PCA, posterior cricoarytenoid), adductor (CT, cricothyroid) and limb (EDL, extensor digitorum longus) muscles in a mouse model of ALI.

**Methods:**

Escherichia coli lipopolysaccharides were instilled into the lungs of adult male C57Bl6J mice (ALI mice). Limb and intrinsic laryngeal muscles were analyzed for fiber size, type, protein expression and myosin heavy chain (MyHC) composition by SDS-PAGE and mass spectroscopy.

**Results:**

Marked muscle atrophy occurred in the CT and EDL muscles, while the PCA was spared. The E3 ubiquitin ligase muscle ring finger-1 protein (MuRF1), a known mediator of limb muscle atrophy in this model, was upregulated in the CT and EDL, but not in the PCA. Genetic inhibition of MuRF1 protected the CT and EDL from ALI-induced muscle atrophy. MyHC-Extraocular (MyHC-EO) comprised 27% of the total MyHC in the PCA, distributed as hybrid fibers throughout 72% of PCA muscle fibers.

**Conclusion:**

The vocal cord abductor (PCA) contains a large proportion of fibers expressing MyHC-EO and is spared from muscle atrophy in ALI mice. The lack of MuRF1 expression in the PCA suggests a previously unrecognized mechanism whereby this muscle is spared from atrophy. Atrophy of the vocal cord adductor (CT) may contribute to the impaired voice and increased aspiration observed in ICU survivors. Further evaluation of the sparing of muscles involved in systemic wasting diseases may lead to potential therapeutic targets for these illnesses.

## Introduction

The acute respiratory distress syndrome (ARDS), also formerly referred to as acute lung injury (ALI) [1] is a common condition marked by the acute onset of pulmonary inflammation and respiratory failure that effects approximately 200,000 patients per year in the United States alone [2]. It is caused by a variety of insults including pneumonia, aspiration, sepsis and trauma [3]. As mortality from ARDS has decreased over time, there has been an increased recognition of the systemic and long-term sequelae of ARDS in survivors. A common complication of ARDS is skeletal muscle weakness, referred to as intensive care unit acquired weakness (ICUAW), which is present in 25 to 50% of ARDS patients and is independently associated with mortality and long-term morbidity in survivors [4], [5], [6]. While no animal model can fully replicate the complex pathophysiology of ARDS in humans, instillation of lipopolysaccharides into the lungs (IT-LPS) of mice is a common model which meets all of the relevant criteria for a mouse model of ALI/ARDS [7]. For the purposes of this manuscript, we consider ALI and ARDS as the same entity and will refer to the IT-LPS mice as ALI mice.

The intrinsic laryngeal muscles play important roles in initiation of and recovery from ARDS. Silent aspiration of colonized oropharyngeal secretions into the lung is the primary mechanism for developing pneumonia. Following ARDS and other critical illnesses requiring mechanical ventilation, patients often have difficulty with both swallowing and voice [8], [9]. It is unclear if these problems are primarily due to mechanical damage from endotracheal tube placement, muscle disuse, or from the systemic muscle wasting associated with critical illness. Additional observations in patients and animal models of critical illness suggest that muscles of the head and neck may hold clues to the pathophysiology of ICUAW. In critically ill humans, one cardinal feature of ICUAW is that the facial muscles are spared [10]. In a porcine critical illness model, the masticatory muscle is relatively spared [11], [12].

The intrinsic laryngeal muscles are groups of pairs of muscles, characterized by small fiber size and fast twitch velocity [13], [14], [15] that play important roles in phonation, respiration and swallowing. Presumably due to the specificity of the intrinsic laryngeal muscles in their anatomical and physiological properties, other investigators have found that the laryngeal muscles are spared or less affected in many muscle wasting disorders. For example, the intrinsic laryngeal muscles display less pathology than limb muscles in dystrophic mouse models including the Duchenne muscular dystrophy (*mdx*) mouse and the congenital muscular dystrophy type 1A mouse [16], [17], [18], [19].

The posterior cricoarytenoid (PCA) muscle abducts the vocal cords while the thyroarytenoid (TA) and cricothyroid (CT) adduct the vocal cords and tenses the focal cords to aid in phonation and airway protection during swallowing [20]. These laryngeal muscles are the most commonly studied among the intrinsic laryngeal muscles in rodents [13], [21]. As the PCA and CT muscles are primarily type II, fast twitch muscles, we chose to compare their response to a primarily type II, fast twitch limb muscle, the extensor digitorum longus (EDL). As we have previously shown that type II muscle fibers are primarily affected in ALI mice [22], we hypothesized that the laryngeal muscles and EDL would be similarly affected.

Muscle ring finger-1 (MuRF1, gene name-*Trim 63*) and atrogin1 (gene name-*MAFbx*) are E3 ubiquitin ligases that were first identified following transcript profiling in fasting and immobilization models of rodent muscle atrophy [23], [24]. We and others have shown that MuRF1 and atrogin1 are expressed in the muscles of ALI mice and critically ill humans [22], [25], [26], [27], [28], [29]. MuRF1, but not atrogin1 is responsible for the limb muscle atrophy that occurs in the early phase of muscle wasting (Days 1–4) in ALI mice [22]. Our aim in this study was to evaluate the intrinsic laryngeal muscles in ALI mice in order to examine mechanisms underlying the vocal morbidity and increased aspiration observed in survivors of critical illness.

## Materials and Methods

### Animals

Two month old male wild type (WT) C57BL/6 (The Jackson Laboratory, Bar Harbor, ME) or MuRF1 knock out (KO) mice comprised of two groups: SHAM and ALI. Mice were anesthetized with an intraperitoneal (i.p.) injection of 150 µg/g ketamine and 15 µg/g xylazine and the trachea exposed. *Eschericia coli* lipopolysaccharide (LPS) (O55:B5 L2880, lot 111M4035V, Sigma-Aldrich, St. Louis, MO) (ALI mice) at 3 µg/g mouse or an equivalent volume of sterile water (SHAM mice) was instilled intratracheally using a 20-gauge catheter as previously described [22]. Neither the orotracheally inserted catheter nor the LPS has contact with the CT or PCA muscles and LPS is injected distal to these muscles, directly into the lungs. All mice were maintained on a 12-hour light/dark cycle and given *ad libitum* access to food and water. All procedures carried out in these experiments were approved by the Institutional Animal Care and Use Committee of Wake Forest University.

### Lung and Bronchoalveolar Lavage (BAL) Collection and Analysis

Three days following ALI or SHAM conditions, mice were euthanized and the lung bronchoalveolar lavage fluids were collected for analysis as previously described [30]. Lung was dissected, fixed in 4% formalin, embedded with paraffin and sectioned for H&E staining.

### Muscle Collection

Under a stereomicroscope (Nikon, SMZ645), the whole larynx was dissected and bilateral PCA, CT and EDL muscle were isolated. The muscles from one side were embedded in OCT (Tissue-Tek, Torrance, CA), quickly frozen in liquid nitrogen, and stored at −80°C for ATPase and H&E staining. The muscles from the other side were immediately frozen in the liquid nitrogen and stored at −80°C.

### Adenosine 5′-triphosphate Disodium Salt Hydrate (ATPase) Staining

Frozen sections (12 µm) from the middle compartment of each muscle were mounted on glass slides for ATPase staining as described before [31], [32]. Briefly, slides were incubated in a 0.01 M ATP (Sigma, St. Louis) solution containing 0.1 M glycine/NaCl with 0.75 M CaCl_2_, pH 10.2, at 37°C for 15 min and then in 2% CoCl_2_ for 5 min. Color was developed in ammonium sulfide solution (1∶50) for 20 seconds. Slides were mounted on glycerin jelly, observed under a microscope, and imaged using TSView 7.1 (Tucsen Imaging Technology, Elmsford, NY). Images of multiple muscle fields of each muscle were taken at 40X magnification and composed as a single cross-section using Adobe Photoshop CS3 (New York, NY). From these images, number of muscle fibers was counted, cross sectional area (CSA) of the mid-section of the muscle was measured and CSA of individual muscle fibers were measured. Each fiber in each muscle was counted or measured.

### Western Blot

To prepare whole muscle lysate, each muscle was homogenized in 50µl laemmli sodium dodecyl sulfate-polyacrylamide gel electrophoresis (SDS-PAGE) sample buffer containing 2% SDS and heated at 95°C for 5 min. Insoluble material was removed by centrifugation, and the soluble supernatants saved as whole cell lysis. Protein concentration was determined by Bio-Rad DC assay (Hercules, CA, USA). For immunoblots, 15 µg protein from each preparation was used. SDS-PAGE was conducted using a 4.5% stacking gel with a 10% resolving gel in a Mini-Protean gel system (BioRad Laboratories, Hemel-Hemptstead, Herts., UK) as described before [33]. The proteins were transferred to nitrocellulose membranes (Amersham, Arlington Heights, IL, USA), blocked with 5% nonfat dry milk with 0.1% Tween in TBS and incubated overnight with MuRF1 (AF5366 R&D Systems, Minneapolis, MN, USA, 1∶1000) and GAPDH (Life Technologies, Grand Island, NY, 1∶3000) for primary antibody incubation. The blots were then incubated with anti-goat or anti-mouse IgG horseradish peroxidase-conjugated antibodies (GE Healthcare, Piscataway, NJ, USA) at a 1∶5000 dilution at room temperature for 1 h. Peroxidase activity was revealed with the Amersham ECL plus western blot detection reagents (GE Healthcare, Piscataway, NJ). Data are representative of three independent experiments. Band intensity was measured using the Kodak Gel Doc imaging system (Carestream Health, Inc, Rochester, NY).

### RNA Extraction, Reverse Transcription, and qPCR

Total RNA was extracted from the whole muscles using Trizol reagent (Invitrogen) following the user manual. Gene expression was analyzed by quantitative real-time PCR (qPCR) using 7500 Fast Real-Time PCR Systems (Applied Biosystems, Foster City, CA, USA). SensiFAST Probe Lo-ROX One-Step Kit and TaqMan primer/probes for MuRF1, atrogin1 and GAPDH genes were purchased from Bioline (Taunton, MA, USA) and Applied Biosystems (Foster City, CA, USA). To determine MuRF1, atrogin1, or GAPDH tissue expression levels, 15 ng of total RNA was added to the PCR reaction tube with a mixture of SensiFAST Probe One-Step mix, Taqman primer/probes, reverse transcriptase and RiboSafe RNAse Inhibitor in a total reaction volume of 25 µl. The PCR parameters were 45°C, 10 min×1 cycle; 95°C, 2 min×1 cycle; 95°C, 15 s, and 60°C, 1 min×40 cycles.

### Analysis of Myosin Heavy Chain (MyHC) Composition in the Whole Muscle and Single Muscle Fiber

MyHC isoforms were separated using SDS-PAGE and 0.6 µg protein from each preparation for western blot was used. SDS-PAGE for MyHC detection was conducted using gel slabs (0.75 mm thick) consisted of 8% separating gel and 4% stacking gel (49∶1 acrylamide-bisacrylamide). All gels were made from the same stock solutions, and all chemicals were of electrophoresis grade. Electrophoresis was performed using a vertical slab gel unit (Bio-Rad Mini-Protean Tetra II, Bio-Rad Laboratories) run at 140 V for 7.5 h at 4°C. Separating gels were silver-stained to visualize the MyHC bands [34] using the Silver Stain Plus Kit (Bio-Rad Laboratories). Mouse skeletal muscle MyHC standards were prepared from pooled muscle (EDL, soleus and diaphragm) samples and run on each gel to verify separation of all MyHC isoforms. Images of silver-stained gels were obtained using an AGFA Duoscan HiD scanner (AGFA Corporation, Ridgefield Park, NJ). The integrated density of individual bands, identified by mass spectrometry (described below) was analyzed and quantified by Image J (1.42q, National Institutes of Health, USA). The composition (%) each MyHC isoform in each muscle was calculated as the integrated density of each band*100/sum of integrated densities of all bands (MyHC isforms) present within the muscle.

Single fiber muscle MyHC analysis was performed as described previously [32]. Briefly, freshly dissected PCA muscle was rinsed in a 4°C relaxing solution containing 7.0 mM EGTA, 14.5 mM creatine phosphate, 20.0 mM imidazole, 4 mM Mg2+-ATP, and 1 mM free Mg2+, transferred to a skinning solution containing 50% glycerol and 50% relaxing solution at 4°C for 24 hours and subsequently stored at −20°C before single fiber isolation. Single fibers were then placed in the Western blot sample buffer as above. MyHC isforms were separated by SDS-PAGE and silver-stained to visualize the MyHC bands. Fiber types by MyHC isoform were quantified as a proportion of the total (80) fibers run.

### Mass Spectrometry Analysis

The corresponding protein bands on the SDS-PAGE gel were excised, chopped into small pieces and transfer to 1.7 ml Maxium-recovery microcentrifuge tubes and subjected to in-gel trypsin digestion. In brief, the gel pieces were destained by 25 mM ammonium bicarbonate in 50% acetonitrile. The proteins in the gel pieces were reduced by dithiothreitol (DTT), alkylated by iodoacetamide (IAA), and then subjected to overnight trypsin (working concentration 10 ng/µL) digestion at 37°C. Tryptic peptides were extracted, lyophilized resuspended in 40 µL of 5% formic acid and further processed on C18 resin, using handmade StageTips [35]. Peptides were eluted with 5% formic acid, 50% acetonitrile, lyophilized with a speed-vac, reconstituted in 0.1% trifluoroacetic acid, 2% acetonitrile, and subjected to LC-MS/MS analysis. LC/MS/MS analyses were performed on a Thermo Scientific LTQ Orbitrap XL (Thermo Scientific) with a Finnigan Nanospray II electrospray ionization source. Peptides were injected onto a 75 µm × 150 mm BEH C18 column (particle size 1.7 µm, Waters) and separated using a Waters nano ACQUITY Ultra Performance LC™ (UPLC™) System (Waters, Milford, MA). The LTQ Orbitrap XL was operated in the data dependent mode using the TOP10 strategy [36]. In brief, each scan cycle was initiated with a full MS scan of high mass accuracy [375–1800 m/z; acquired in the Orbitrap XL at 6 × 10^4^ resolution setting and automatic gain control (AGC) target of 106], which was followed by MS/MS scans (AGC target 5000; threshold 3000) in the linear ion trap on the 10 most abundant precursor ions. Selected ions were dynamically excluded for 30 s. Singly charged ions were excluded from MS/MS analysis. MS/MS spectra were searched against a composite database containing the IPI Homo sapiens (human) protein sequences and their reverse sequences using Proteome Discover 1.3 (Thermo). Search parameters allowed two missed tryptic cleavages, a mass tolerance of ±10 ppm for precursor ion, and a mass tolerance of ±0.8 Da for product ion, a static modification of 57.02146 Da (carboxyamidomethylation) on cysteine, and a dynamic modification of 15.99491 Da (oxidation) on methionine. Search results were filtered under 1% false discovery rate (FDR) criteria.

### Data Analysis

Variables are expressed as mean ± standard error. Data were analyzed using an independent two-tailed t-test or two-way ANOVA with condition (SHAM and ALI) and muscle type (PCA, CT, and EDL) as grouping variables. Post-hoc analysis was performed using a t-test with the Bonferroni adjustment method. P values less than 0.05 were considered statistically significant.

## Results

### Acute Lung Injury Mouse Model

**Figure 1:**
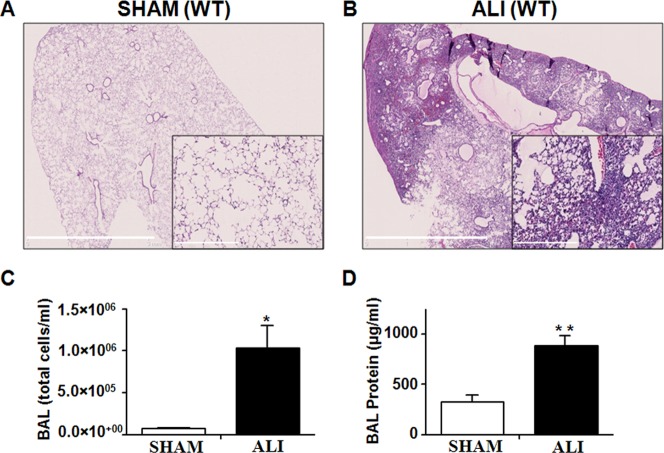
Lung inflammation in ALI mice. A. H&E stained SHAM lungs at day 3 reveal normal lung alveolar architecture at low and high (inset) magnification. B. ALI lungs demonstrate “hepatization” of the lung, with alveolar consolidation, interstitial thickening and hemmorrhage. The degree of lung injury is quantified with BAL (C) total cell counts and (D) total protein which are increased in the lung of the ALI compared to SHAM mice. *p = 0.0003, **p = 0.008; n = 6/group.

As previously reported [22], [30] and shown in Figure 1, intratracheal instillation of LPS caused profound lung inflammation at 3 days with cellular infiltration, hemorrhage, consolidation of alveolar spaces and thickening of the interstitial space (Figures 1B). Day 3 is the timepoint of peak lung injury and the peak of the early muscle atrophy program in this model [22]. Lung histology was normal in SHAM mice (Figure 1A). BAL fluid analysis revealed this injury with increased total cells (Figure 1C) and protein (Figure 1D) in the alveolar space of ALI compared to SHAM mice.

### The PCA is Spared from Muscle Atrophy in ALI Mice

**Figure 2:**
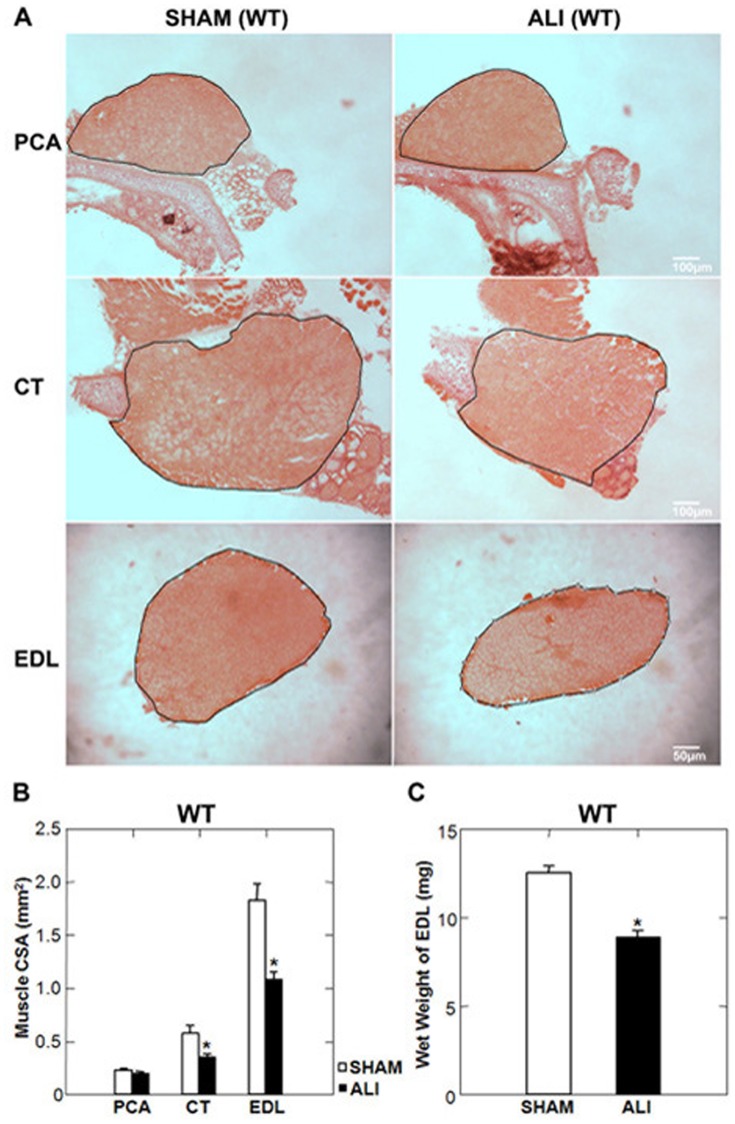
The PCA muscle is spared from atrophy in WT ALI mice. A. Low power H&E stained mid-sections of the PCA, CT and EDL muscles of WT SHAM and ALI mice. The black line represents the border of the muscle boundary. B. CT and EDL muscle cross sectional areas (CSA) of ALI mice are reduced compared to SHAM, while the CSA of the PCA muscle is unchanged. C. The wet weight of the EDL is also reduced in ALI versus SHAM mice. *p<0.05; n = 5/group.

The mass of the EDL and the cross sectional area (CSA) at the midsection of both the EDL and CT were reduced in WT ALI compared to WT SHAM mice. The PCA muscle CSA was unchanged between WT SHAM and WT ALI mice (Figure 2). Due to the small size and difficulty of consistently removing the entire muscle, we were not able to accurately measure the mass of the laryngeal muscles. We observed no difference in the total number of fibers between SHAM and ALI mice in any muscle (Supplemental Figure S1A&B) suggesting that myofiber atrophy, not fiber loss was the etiology for reduced muscle mass and CSA. We also found no evidence of fiber type switching using the ATPase method of staining to identify fiber types (Supplemental Figure S1A,C&D).

**Figure 3:**
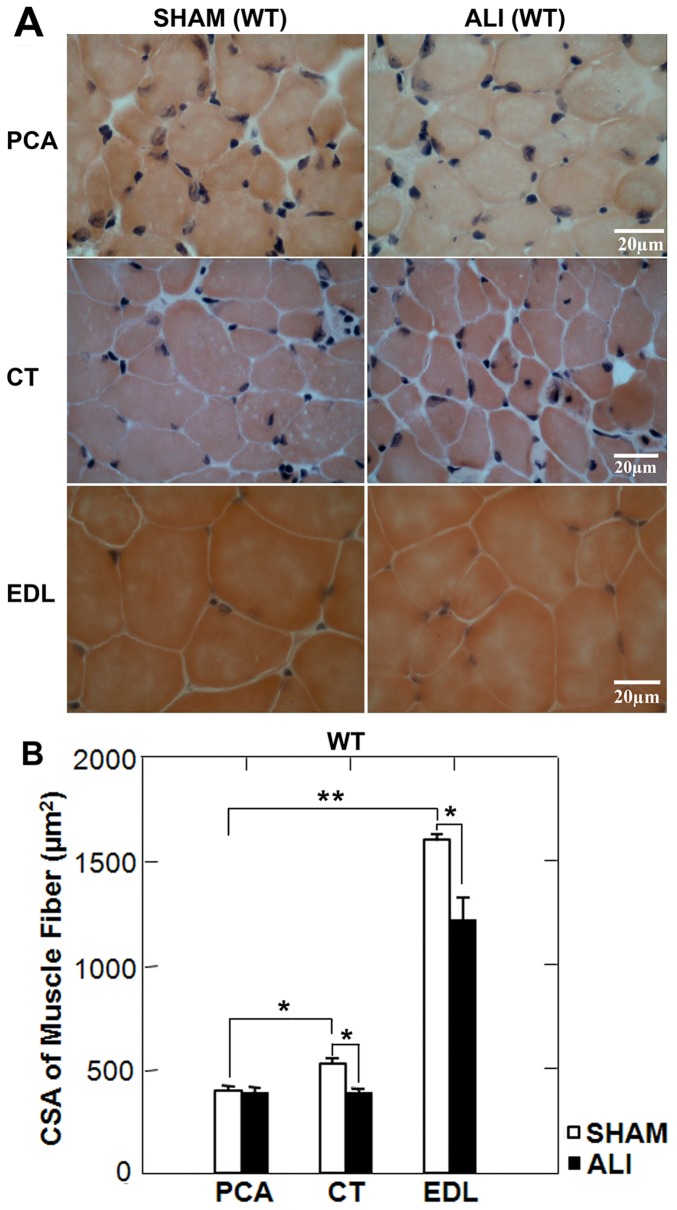
PCA myofibers are spared from atrophy in WT ALI mice. A. High power H&E stained sections of the PCA, CT and EDL muscles of WT SHAM and ALI mice. B. Mean myofiber CSA of SHAM and ALI PCA, CT, and EDL myofibers reveal that SHAM PCA fibers are smaller than either CT or EDL myofibers and that PCA myofibers are spared from ALI induced myofiber atrophy. *p<0.05, **p<0.001; n = 5/group.

Morphometric analysis of H&E stained sections of the EDL, CT and PCA demonstrated that WT SHAM PCA myofibers were smaller than either the CT or EDL SHAM myofibers. WT ALI myofibers of the EDL and CT exhibited evidence of myofiber atrophy, while the myofibers of the PCA exhibited no evidence of myofiber atrophy (Figure 3).

### MuRF1 and Atrogin1 Expression in Limb and Laryngeal Muscles of WT ALI Mice

**Figure 4:**
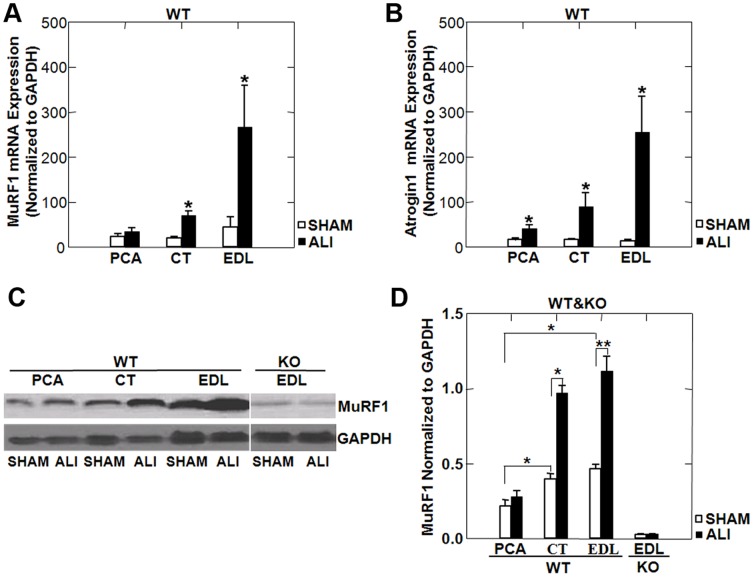
MuRF1 and atrogin1 mRNA and protein expression in WT ALI mice. MuRF1 (A) and atrogin1 (B) mRNA normalized to GAPDH in the PCA, CT and EDL of SHAM and ALI mice reveal upregulation of atrogin1 in all muscles of ALI mice, but upregulation of MuRF1 only in the CT and EDL, not the PCA of ALI mice. Muscle lysates probed for MuRF1 protein, normalized to GAPDH (C), and quantified by densitometry (D) reveal reduced basal expression of MuRF1 in the PCA of SHAM mice and upregulation of MuRF1 protein in the CT and EDL, but not the PCA of ALI mice. *p<0.05, **p<0.01; n = 3/group.

As we have previously shown that MuRF1 expression is critical for the muscle atrophy occurring in the limb muscles of ALI mice [22], we probed SHAM and ALI EDL, CT and PCA muscles for MuRF1 and atrogin1 mRNA expression and MuRF1 protein expression. We found increased atrogin1 mRNA in all three muscles in ALI versus SHAM mice (Figure 4B) while MuRF1 was upregulated only in the CT and EDL, but not in the PCA (Figure 4A). Representative western blots (Figure 4C) and densitometry (Figure 4D) show that basal levels (in SHAM mice) of MuRF1 protein were increased in the CT and EDL compared to the PCA. MuRF1 protein was also upregulated under ALI conditions in the CT and EDL, but not in the PCA.

### MuRF1 KO ALI Mice are Protected from Laryngeal and Limb Muscle Atrophy

**Figure 5:**
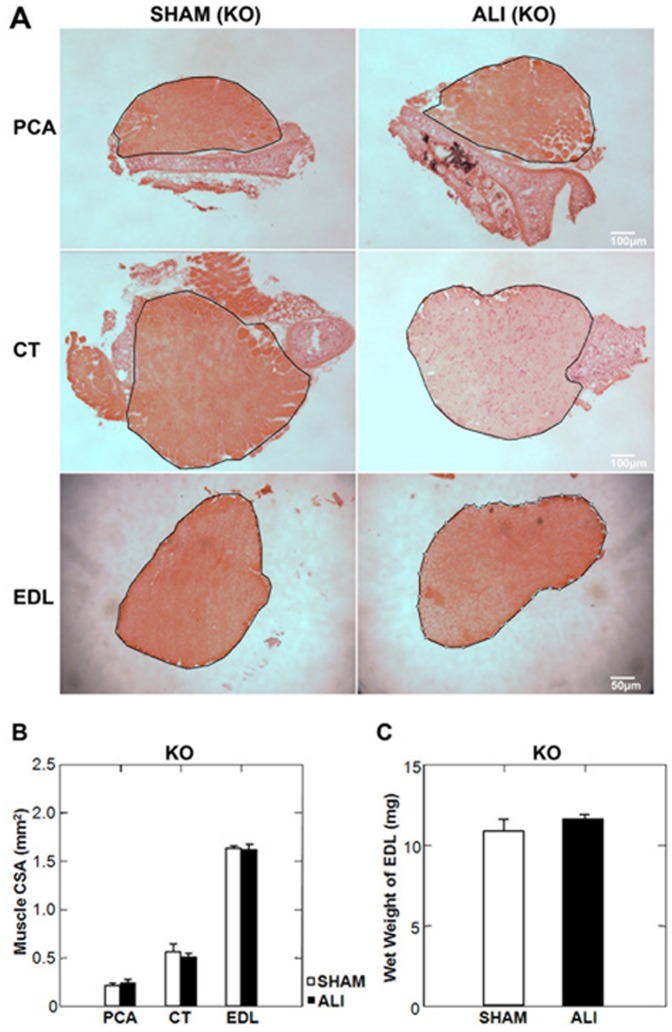
MuRF1 KO ALI mice are spared from limb and laryngeal muscle atrophy. A. Low power H&E stained mid-sections of the PCA, CT and EDL muscles of KO SHAM and ALI mice. The black line represents the border of the muscle boundary. B. No change in limb or laryngeal muscle CSA was observed in MuRF1 KO ALI mice at day 3. C. The wet weight of the EDL in MuRF1 KO ALI mice is unchanged compared to MuRF1 KO SHAM mice. n = 3/group.

**Figure 6:**
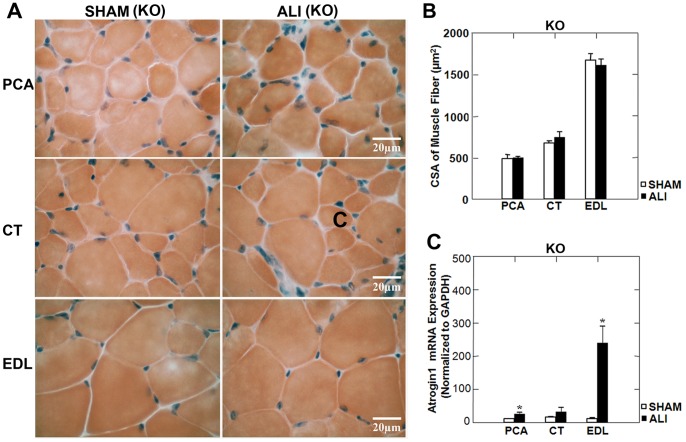
MuRF1 expression is necessary for limb and larygeal myofiber atrophy in ALI mice. A. High power H&E stained sections of the PCA, CT and EDL muscles of MuRF1 KO SHAM and ALI mice (B) reveal no change in myofiber CSA under ALI conditions. Despite no evidence of myofiber atrophy in any muscle, atrogin1 mRNA (C) was upregulated in the PCA and EDL muscles of MuRF1 KO ALI versus SHAM mice. *p<0.05, n = 3/group.

Lung injury at day 3 in MuRF1 KO ALI mice, measured by BAL cell counts was equivalent to that seen in WT ALI mice (Supplemental Figure S2). In contrast to WT mice, PCA, CT and EDL muscles were spared from muscle atrophy under ALI conditions (Figure 5). As in the WT ALI mice, we found no evidence of fiber loss in the MuRF1 KO ALI mice (Supplemental Figure S3). Muscle fiber CSA was not reduced in any muscle in MuRF1 KO ALI versus MuRF1 KO SHAM mice (Figure 6). Despite no evidence of muscle atrophy in the limb and laryngeal muscles of MuRF1 KO ALI mice, we found that atrogin-1 mRNA was upregulated in the PCA and EDL muscles in of MuRF1 KO ALI versus MuRF1 KO SHAM mice (Figure 6C). This supports the evidence that upregulation of atrogin1 mRNA is not sufficient to induce muscle atrophy in this model.

### Identification of Myosin Heavy Chain Isoforms by Mass Spectrometry

**Figure 7:**
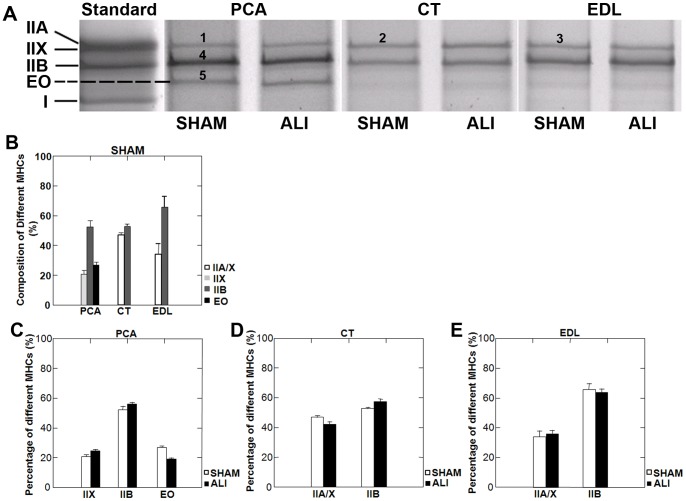
MyHC isoforms in the limb and laryngeal muscles of WT mice. A. Silver stained gels of separated MyHC isoforms of representative SHAM and ALI mice in the PCA, CT and EDL muscles. The PCA muscle lysate included an additional band (band 5), which was not present in the standard, CT or EDL lysates. Mass spectroscopy analysis (see table 1) revealed band 5 to be MyHC-Extraocular (EO) isoform. Distribution of MyHC isoforms (B) in each muscle was quantified by the percentage of integrated density of each bands within each muscle (n = 5/group). No evidence of MyHC isoform switching was present in ALI mice at day 3 in the PCA (C), CT (D) or EDL (E) muscle.

In order to further explain the protection of the PCA from ALI-induced skeletal muscle atrophy, we separated MyHC isoforms on skeletal muscle lysates using SDS-PAGE. Silver staining of the gels detected four bands in the standard sample (a mixture of three muscles: EDL, soleus and diaphragm) lysates. These four bands correspond to the IIA, IIX, IIB and I MyHCs respectively based on our experience and previous literature [37] (Figure 7A). Three bands in the PCA and two in the CT and EDL muscles were detected (Figure 7A). The 3^rd^ band in the PCA (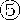 in Figure 7A) was not present in the standard sample. The two bands present in the CT and EDL and the 3 bands present in the PCA were cut from the gel and analyzed by mass spectrometry. Table 1 showed the MyHCs of 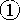-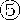 bands marked in Figure 7A. The uppermost band in the PCA muscle (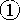 in Figure 7a) contained exclusively type IIX (myosin 1) MyHC while the upper bands in the CT (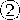 in Figure 7A) and EDL (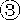 in Figure 7A) muscles contained both IIX (myosin 1) and IIA (myosin 2) MyHC. The band located at position 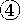 was identified as type IIB (myosin 4) MyHC. The band migrating in between the type IIB and type I bands, which was present only in the PCA (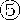 in Figure 7A) was identified as MyHC- Extraocular isoform (myosin, heavy polypeptide 13). MyHC- Extraocular (EO) is encoded by the MYH13 gene and is expressed in human [38] and mouse extraocular muscles [39]. MyHC-EO comprised 27% of the total MyHC was observed in the PCA (Figure 7B). We found no evidence of a MyHC shift between SHAM and ALI conditions in any muscle (Figure 7C–E) by mass spectrometry, confirming the ATPase fiber typing method for evaluation of a fiber type switch (Supplemental Figure S1).

**Table 1:** Summary of MyHC isoforms identified by mass spectrometry.

SDS-PAGE band	Protein IDs	Protein name	Sequence Coverage
**1**	IPI00380896.1	Myosin-1/IIX	7.67
**2**	IPI00380896.1	Myosin-1/IIX	11.74
	IPI00649292.1	Myosin, heavy polypeptide 2/IIA, skeletal muscle, adult	10.62
**3**	IPI00649292.1	Myosin, heavy polypeptide 2/IIA, skeletal muscle, adult	17.68
	IPI00380896.1	Myosin-1/IIX	17.46
**4**	IPI00404837.3	Myosin-4/IIB	28.21
**5**	IPI00468665.2	Extraocular myosin, heavy polypeptide13, skeletal muscle	11.25

**Figure 8:**
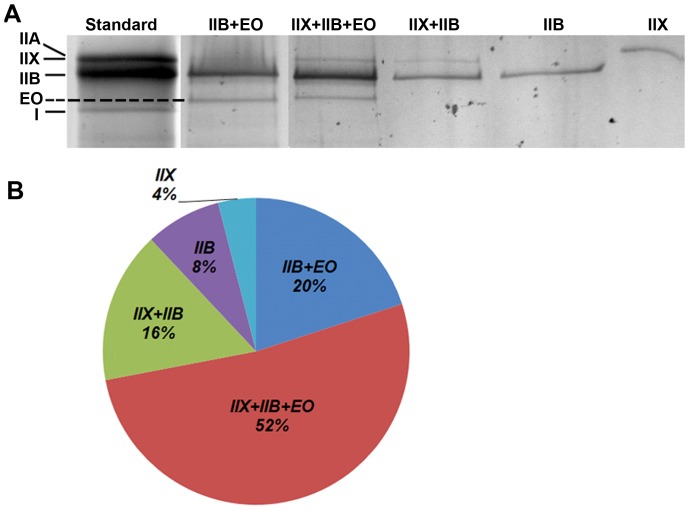
Identification of muscle fiber types in the PCA by MyHC isotyping of single fibers. 80 single fibers from the PCA were dissociated and run in individual lanes and silver stained for identification of MyHC isoforms present within each muscle fiber. A. Representative examples of the differing MyHC isoforms present within single fibers of the PCA muscle. B. Quantification of the percentage of MyHC fiber types present in the PCA muscle revealed 88% of fibers co-expressed multiple MyHC isoforms and 72% of muscle fibers contained MyHC-EO.

Next, we sought to evaluate the individual fiber types present in the PCA. Initial attempts at evaluating the PCA MyHC isotypes by antibody immunofluorescence revealed the presence of extensive hybrid fibers and differential MyHC expression along the length of the fiber with serial sectioning. Due to this and the difficulty in quantifying individual myosin isotypes in hybrid fibers [39], we pursued individual fiber typing by separation of MyHC isotypes in isolated single fibers of the PCA muscle. 80 individual fibers were dissociated from the PCA, and run on SDS-PAGE gels (see Methods). Using this method, we did not find any PCA fibers exclusively expressing only MyHC-EO but found that 72% of the PCA fibers co-express MyHC-EO with IIB or IIX and IIB. Only 12% of fibers were exclusively expressing IIX or IIB (Figure 8).

## Discussion

ICUAW is a common problem occurring in up to 45% of all critically ill patients [40]. The diagnosis of ICUAW in ARDS patients is independently associated with mortality and contributes to long-term morbidity in survivors [5], [41], [42], [43]. Though often overlooked, the laryngeal muscles are important muscles in the pathophysiology of ARDS, as aspiration is a risk factor for ARDS development and speech, swallow and voice impairments often occur in ICU survivors [8], [44].

In our model, limb muscle atrophy occurring 3 days following LPS instillation is the result of lung-induced systemic inflammation. Muscle wasting is likely initiated by one or more systemic inflammatory cytokines leading to MuRF1-mediated muscle atrophy via an NFkB dependent mechanism [22], [26]. This study extends that data by examining the intrinsic laryngeal muscles in ALI mice. The major findings of our current study are as follows: 1) the CT and EDL developed muscle atrophy in WT ALI mice, while the PCA muscle was spared; 2) MuRF1 mRNA and protein of were upregulated in the laryngeal adductor (CT) and limb EDL muscles of ALI mice, but not in the laryngeal abductor (PCA) muscle; 3) the laryngeal and EDL muscles are spared of atrophy in the MuRF1 KO ALI mice despite the upregulation of atrogin1 mRNA; 4) the PCA has a high proportion of hybrid fibers (88%), and 72% of the fibers of this muscle express MyHC-EO.

### The Intrinsic Laryngeal Muscles in ALI Mice

Intrinsic laryngeal muscles are characterized by a higher composition of fast-twitch fibers presumably to fit its functional demands during phonation, respiration and swallowing. These demands include high twitch velocities, while maintaining resistance to fatigue. The PCA functions primarily to abduct the vocal cords, while the CT adducts and tense the vocal cords assisting the vocal fold closure for airway protection. We found that ALI mice developed atrophy of the CT, but not the PCA muscle. The CT muscle atrophied in ALI mice independent of tracheal tube placement (as SHAM mice also had tracheal tubes transiently placed), suggesting that laryngeal muscle atrophy, as well as limb muscle atrophy occur via lung-induced systemic wasting in this model. Work is ongoing in our labs to understand the specific systemic mediators of this process. While we were not able to measure laryngeal muscle function in this study, we theorize that this pattern of atrophy may lead to impaired voice quality and a predisposition to aspirate when swallowing. Future studies in our lab will measure the functional aspects of laryngeal muscle atrophy in ALI mice.

### The Presence MyHC-Extraocular Isoform in the PCA Muscle

We found that 27% of the total MyHC in the PCA muscle was MyHC-EO (Figure 7) and that 72% of PCA muscle fibers have MyHC-EO present (Figure 8). To our knowledge, fiber type composition of the PCA in adult mice has not been performed. MyHC-EO is present in the extraocular muscles in rodents and humans [38], [39]. Previous investigators have shown that the extraocular muscles are more diverse in their expression of MyHC when compared to limb muscles. For example, Zhou et al found that adult C57Bl6j mice expressed 8 subtypes of MyHC, with MyHC-EO (MYH13) comprising 4% of the total MyHC in the whole extraocular muscle. In addition, the extraocular muscles have a high prevalence of hybrid fibers co-expressing multiple types of MyHC along the length of the myofiber [39], presumably facilitating the complicated biomechanical needs of these muscles. We found significant difficulty in staining the PCA with MyHC isotype specific antibodies, with weak staining and inconsistent results on serial sectioning. This is likely due to the high proportion of hybrid fiber and possible differential MyHC expression along the length of the muscle fiber.

Other investigators have found that the PCA is spared from pathology in dystrophic diseases in rodents and humans [45], [46]. We postulate that the presence of MyHC-EO in the PCA, but not in the CT may contribute to the differential response to ALI induced muscle atrophy in this muscle.

### The Role of MuRF1, Atrogin1 and MyHC-EO in the Laryngeal Muscles of ALI Mice

MuRF1 and atrogin1 gene expression increases in skeletal muscle during a variety of atrophy inducing conditions [47], [48]. While initially thought to possibly be redundant E3 ligases, emerging evidence suggest that atrogin1 and MuRF1 may mediate the muscle atrophy differentially. The contractile proteins targeted for ubiquitination by these E3 ligases differ [49], [50], [51]. Evidence also exists for differential regulation under differing disease states. For instance, the muscle atrophy in chronic obstructive pulmonary disease (COPD) involves the atrogin1 mRNA up-regulation without changes in MuRF1 levels [48]. We have shown that although mRNA expression of both the MuRF1 and atrogin1 was increased in ALI mice, only MuRF1 was responsible for the muscle atrophy [22]. In this study, we found that the PCA muscle was spared from ALI induced atrophy, apparently through the absence of MuRF1 upregulation under ALI conditions.

MyHC is a primary target for MuRF1 mediated ubiqutination and degradation [49], though its specificity for differing MyHC isoforms has not been studied. Previous investigations have shown that MuRF1 is expressed in muscle at basal levels under normal physiologic conditions primarily in type II limb myofibers [52]. We hypothesized that MuRF1 expression would be high in the fast-twitch intrinsic laryngeal muscles but found that basal MuRF1 expression was lower in the PCA when compared to the CT or EDL.

Since MyHC-EO is not purely expressed in single fibers, its properties have been difficult to study, until a recent report that investigates this by expressing pure MyHC-EO in murine C2C12 cells. This study confirmed the ultra fast kinetics of MyHC-EO [53], though it also has functional characteristics similar to the slower MyHC isoforms [54]. We hypothesize that the presence of MyHC-EO may limit both the basal expression and upregulation of MuRF1 under atrophy inducing conditions, protecting the PCA from atrophy. It is also possible that MuRF1 is unable to ubiquinate MyHC-EO. Future studies are needed to further examine this relationship.

Other characteristics of the PCA, including its neuromuscular junction structure or satellite cell regenerative potential may also contribute to its protection from atrophy [21], [55]. A better understanding of this process could lead to targeted treatment of muscle atrophy in systemic muscle wasting diseases.

## Conclusion

The data from this study provides preclinical evidence into laryngeal muscle dysfunction in ALI pathophysiology and sheds new light on the mechanisms underlying this process. A better understanding of the sparing of the PCA in ALI mice will accelerate the mechanistic understanding of ICUAW in order to improve the lives of ICU survivors. This data also highlights the unique properties of muscle fibers expressing MyHC-EO and provides a potential mechanism underlying the sparing of the PCA muscle in this and other muscle wasting conditions.

## Supporting Information

Figure S1 **ATPase staining of limb and laryngeal muscles of WT SHAM and ALI mice.** A. ATPase staining at pH 9.8 revealed two easily discernible color gradations, dark (presumably IIA/X) and intermediate (presumably IIB) fibers in each of the muscles of SHAM and ALI WT mice. (We determined in Figure 7, 8 and Table 1 that the PCA is composed of a large proportion of hybrid fibers expressing MyHC-EO). B. The total number of myofibers between SHAM and ALI mice was unchanged. C. Quantification of dark and intermediate fibers revealed no evidence of a fiber type switch by the ATPase method in any muscle in ALI mice.
(TIF)

Figure S2 **Lung inflammation in WT and MuRF1 KO SHAM and ALI mice.** Lung inflammation at day 3 in WT and MuRF1 KO ALI mice, measured by BAL total cell counts, was similarly increased compared to SHAM WT or SHAM MuRF1 KO mice.
(TIF)

Figure S3 **ATPase staining of limb and laryngeal muscles of MuRF1 SHAM and ALI mice.** A. ATPase staining at pH 9.8 revealed two easily discernible color gradations, dark (presumably IIA/X) and intermediate (presumably IIB) fibers in each of the muscles of SHAM and ALI MuRF1 KO mice. B. The total number of myofibers between SHAM and ALI MuRF1 KO mice was unchanged. C. Quantification of dark and intermediate fibers revealed no evidence of a fiber type switch by the ATPase method in any muscle in MuRF1 KO ALI mice.
(TIF)
