# Supplementary figures and images for: The Posterior Cricoarytenoid Muscle Is Spared from MuRF1-Mediated Muscle Atrophy in Mice with Acute Lung Injury
Source: PLoS One. 2014 Jan 31;9(1):e87587. doi: 10.1371/journal.pone.0087587 (PMC3909200; doi:10.1371/journal.pone.0087587)

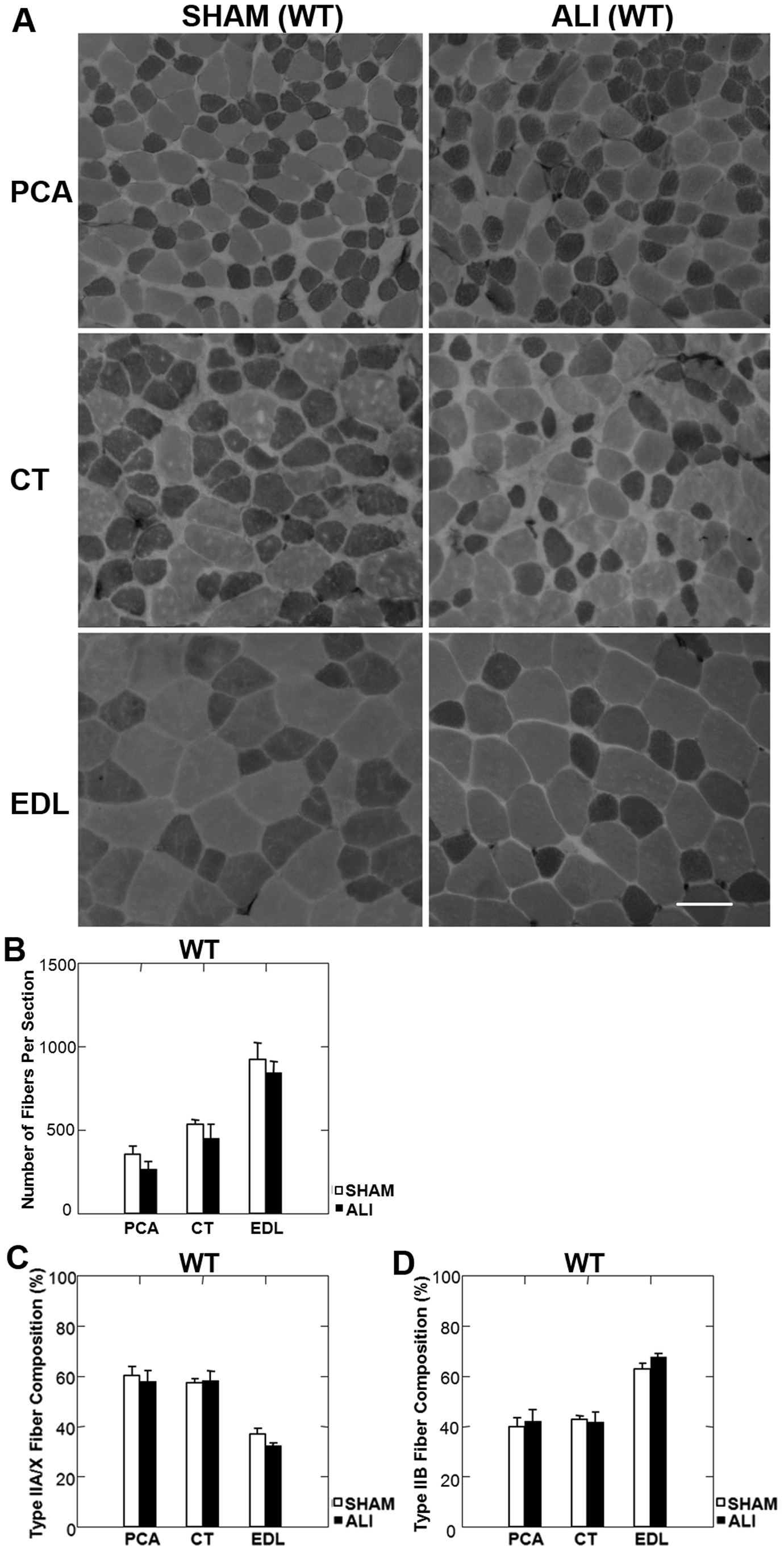

Supplement: Figure S1 — ATPase staining of limb and laryngeal muscles of WT SHAM and ALI mice. A. ATPase staining at pH 9.8 revealed two easily discernible color gradations, dark (presumably IIA/X) and intermediate (presumably IIB) fibers in each of the muscles of SHAM and ALI WT mice. (We determined in Figure 7, 8 and Table 1 that the PCA is composed of a large proportion of hybrid fibers expressing MyHC-EO). B. The total number of myofibers between SHAM and ALI mice was unchanged. C. Quantification of dark and intermediate fibers revealed no evidence of a fiber type switch by the ATPase method in any muscle in ALI mice. (TIF) [file pone.0087587.s001.tif]

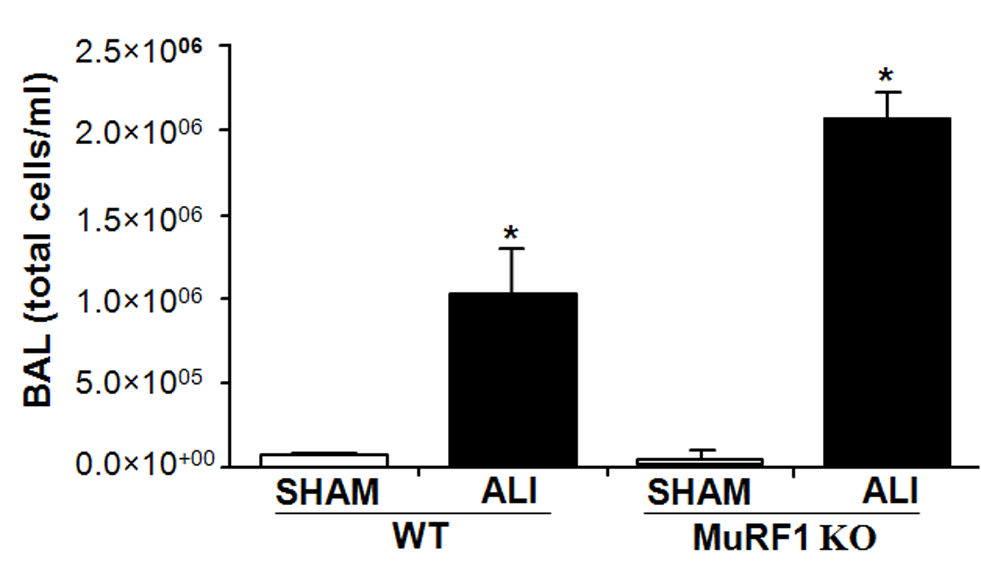

Supplement: Figure S2 — Lung inflammation in WT and MuRF1 KO SHAM and ALI mice. Lung inflammation at day 3 in WT and MuRF1 KO ALI mice, measured by BAL total cell counts, was similarly increased compared to SHAM WT or SHAM MuRF1 KO mice. (TIF) [file pone.0087587.s002.tif]

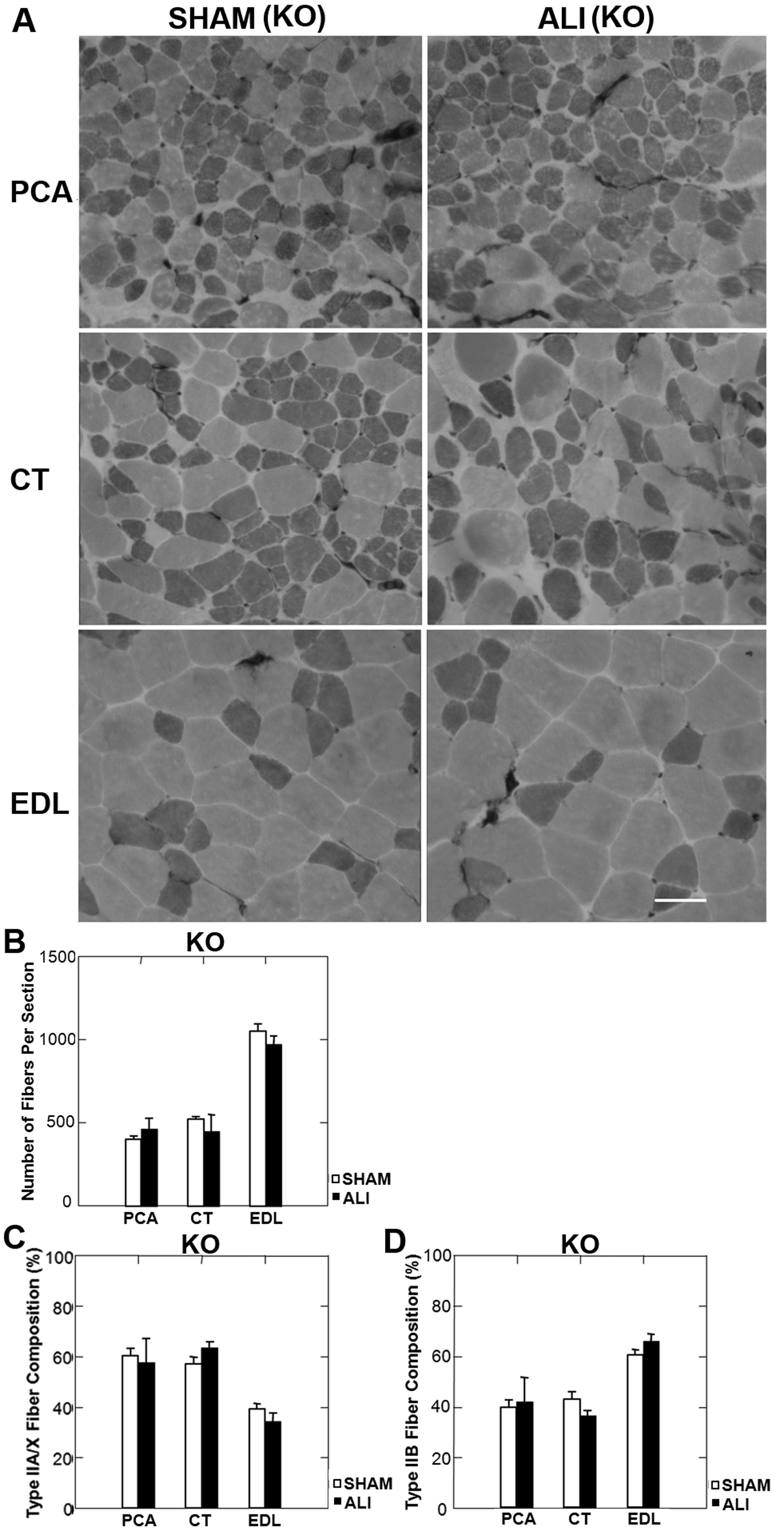

Supplement: Figure S3 — ATPase staining of limb and laryngeal muscles of MuRF1 SHAM and ALI mice. A. ATPase staining at pH 9.8 revealed two easily discernible color gradations, dark (presumably IIA/X) and intermediate (presumably IIB) fibers in each of the muscles of SHAM and ALI MuRF1 KO mice. B. The total number of myofibers between SHAM and ALI MuRF1 KO mice was unchanged. C. Quantification of dark and intermediate fibers revealed no evidence of a fiber type switch by the ATPase method in any muscle in MuRF1 KO ALI mice. (TIF) [file pone.0087587.s003.tif]
